# Use of a New Comprehensive Insurance Benefit for Smoking-Cessation Treatment

**Published:** 2005-09-15

**Authors:** Marguerite E Burns, Marjorie A Rosenberg, Michael C Fiore

**Affiliations:** Center for Tobacco Research and Intervention and Department of Population Health Sciences, University of Wisconsin Medical School; Department of Actuarial Science, Risk Management and Insurance, University of Wisconsin School of Business and Department of Biostatistics and Medical Informatics, University of Wisconsin Medical School, Madison, Wisc; Center for Tobacco Research and Intervention and Department of Medicine, University of Wisconsin Medical School, Madison, Wisc

## Abstract

**Introduction:**

Uncertainty about levels of employee use of an insurance benefit for smoking-cessation treatment has presented a barrier to employers considering the adoption of such coverage. This study examined self-reported awareness and use of a new insurance benefit for smoking-cessation treatment among a sample of Wisconsin state employees, retirees, and adult dependents.

**Methods:**

We evaluated the self-reported use of insurance coverage for smoking-cessation treatment during the first 2 years of its availability to the Wisconsin state employee, retiree, and adult dependent population. We conducted analyses of responses to smoking-related questions in 2001 and 2002 cross-sectional surveys of insured state employees, retirees, and adult dependents, weighted to represent this population.

**Results:**

In 2002, benefit use among smokers aware of the benefit was 39.6%, and benefit use among smokers unaware of the benefit was 3.5%. Only 27.4% of smokers were aware of the benefit in 2002; use among all smokers was 13.6%. Of all smokers, 30.4% used smoking-cessation treatment medication (over-the-counter or covered) in 2002. Smoking prevalence was 15.6% in 2001 and 13.2% in 2002.

**Conclusion:**

In an educated employee population, self-reported smoking-cessation treatment benefit use was modest among all smokers during its first 2 years of availability. Benefit awareness was low in this educated population, which may help explain low use rates, particularly given the 30% of all smokers who attempted to quit smoking with the help of smoking-cessation treatment medication. These data provide use-rate estimates for states contemplating adoption of an evidence-based smoking-cessation treatment benefit.

## Introduction

As part of a comprehensive tobacco-control strategy, public health experts recommend insurance coverage for evidence-based smoking-cessation treatments (SCTs) such as smoking-cessation medications approved by the Food and Drug Administration (FDA) (1-3). These medications can double or triple the likelihood of quitting smoking compared with quitting without such treatment (3). Moreover, insurance coverage for evidence-based SCTs has been shown to reduce smoking rates in insured populations (4,5). Although some health plans have adopted these public health recommendations, a sizeable gap remains between the recommended availability and the actual availability of such coverage. For example, a sample of U.S. health plans reported that 41% of their best-selling commercial health maintenance organization (HMO) products provide coverage for at least one FDA-indicated smoking-cessation medication (6). Coverage among employers varies despite research showing that workplace productivity increases and absenteeism decreases among former smokers compared with smokers (7). Nationally, 23% to 64% of employers provide some form of insurance coverage for SCTs (8,9). Health insurers and employers have offered various explanations for the limited provision of health insurance coverage for SCTs; among these is their uncertainty about the levels of use of such coverage.  

In a series of focus groups, employers cited low benefit-use rates as one of the primary reasons for not covering preventive health services, including SCTs, and questioned the need to provide coverage for services that their employees rarely used (10). Health care purchasers, insurance brokers, and benefits consultants have named a lack of beneficiary demand as one explanation for their limited efforts to negotiate health-behavior change benefits and programs, including SCTs (11). Insurers have also expressed skepticism about member use of SCT coverage and thus the need to cover it. Insurers have asserted that many people quit smoking on their own (12), and that smoking-cessation medication is available over-the-counter (OTC); thus, SCT coverage is redundant (13).

Uncertainty about levels of SCT use poses a second challenge to adopting such coverage: employers and insurers find it difficult to anticipate the cost of SCT coverage. At a minimum, employers require short-term cost estimates for an SCT benefit to make a coverage decision (14). Employers have further indicated that to increase coverage for preventive services such as SCTs, information on coverage costs and potential returns on such an investment is also needed (10).

In recent years, several studies have addressed the extent to which insured individuals use SCT benefits when they are available. In evaluating four SCT-benefit designs on benefit use and smoking outcomes, Curry et al estimated SCT benefit-use rates of 2.4% to 10% among smokers; rates depended upon the benefit design (4). To use the medication component of these benefits, the health plan required smokers to participate in a smoking-cessation counseling program. This requirement may have reduced use of the benefit. If so, the study findings may underestimate population use rates for a pharmacotherapeutic benefit that does not require participation in a counseling program.

In another study, Schauffler et al examined the relationship between SCT coverage, SCT use, and smoking outcomes; they reported that 25% of smokers with SCT coverage used the covered medication (5). The sample included only smokers who were willing to participate in a randomized controlled trial. To the extent that the smokers in the sample were more interested in quitting than a population-based sample of smokers (they volunteered for the study), the results may overestimate SCT benefit-use rates.

In other aspects of the studies discussed above, Schauffler et al and Curry et al showed that providing insurance coverage for SCTs increases treatment use (5) and reduces smoking prevalence within defined populations (4). In contrast, Boyle et al found similar SCT-use rates for smokers with and without SCT insurance coverage; approximately 24% of smokers used bupropion, and 27% used nicotine replacement therapy to make an attempt to quit (15). In the Boyle sample, almost half of smokers reported an interest in quitting within the next 30 days. Because of the interest in quitting among smokers in the sample, the treatment-use rates observed in this population may overestimate rates that an employer or insurer could expect from their total population. For example, in a population-based study, Wewers et al found that approximately 8% of daily smokers planned to make an attempt to quit during the next 30 days. However, this same group of smokers also reported that they had stopped smoking for a day or more during the past 12 months (16). One cannot conclude that the results of these three studies on SCT benefit use will apply to population-based studies.

The purpose of the present population-based study is to evaluate the self-reported use of insurance coverage for SCTs during the first 2 years of its availability to the Wisconsin state employee, retiree, and adult dependent population. The study findings are intended to reduce the uncertainty surrounding what employers, particularly large public employers, can expect when they provide a new SCT benefit.

In January 2001, the Wisconsin Department of Employee Trust Funds (DETF) introduced a health insurance benefit for SCTs for its approximately 183,000 insured employees and their dependents. The DETF required that state employee health insurance plans provide counseling and prescription medication for smoking cessation. (One health plan was exempt from this requirement. Its members, approximately 10% of the total insured population, were excluded from this study.) The benefit included one 3-month course of prescription pharmacotherapy and one office visit for counseling per calendar year. Counseling was not required to obtain pharmacotherapy, and OTC medication was not covered by the benefit. There was no lifetime limit. Table 1 provides a summary of the benefit.

Before January 2001, the availability and scope of coverage for SCTs for Wisconsin state employees, retirees, and adult dependents varied widely. A 1998 survey found that 7 of the 25 Wisconsin state employer-sponsored health plans covered some form of SCT. Significant up-front patient cost sharing was often required, with reimbursement contingent upon the completion of counseling, maintaining abstinence from smoking, or both (13). Thus, the benefit introduced in 2001 expanded the availability of SCTs for the insured state employee, retiree, and adult dependent population.

The DETF notified its employee and retiree populations in October 2000 of the new SCT benefit in the open enrollment materials provided to employees, retirees, and adult dependents. A summary of the SCT benefit was included among other health plan or benefit changes listed in the first two pages of the group health insurance plans and provisions booklet (17).

## Methods

From approximately March through June in both 2001 and 2002, the DETF conducted a computer-assisted telephone-interviewing (CATI) survey of covered state employees, retirees, and adult dependents to assess their experiences and satisfaction with their health insurance plan and health care. The DETF used the adult commercial Consumer Assessment of Health Plans Survey (CAHPS) (18), adding questions on smoking to the CAHPS core instrument for survey years 2001 and 2002. Added questions covered current smoking status, awareness of the SCT insurance benefit, use of the SCT benefit, and in 2002, use of over-the-counter SCTs. We collected sociodemographic data on age, education, and sex.

CATI interviewers progressed through a four-step training process, including 1) learning about the data-collection instrument, 2) mock interviewing, 3) monitoring veteran interviewers, and 4) ongoing performance feedback through the data-collection period. A minimum of 10% of all interviews was monitored by telephone laboratory supervisors.

The sampling frame included all state employee and retiree contracts for individual or family employer-sponsored health insurance in which employees had been in their selected health plan for 12 or more months. In 2001, the sampling frame included 82,984 contracts; in 2002, the sampling frame included 69,600 contracts. The sampling frame was stratified by health insurance plan or carrier (henceforth called *plan*), with 19 plans in 2001 and 22 plans in 2002. The sample size in each stratum was based on two considerations: 1) the estimated number of state employees, retirees, and dependents in each plan and 2) the state's interest in reaching a precision level of ±5% of each plan's population mean for the response to each survey question with 95% confidence. After the sample size by stratum was determined, a random sample of employee and retiree health insurance contracts was selected within each stratum with a goal of achieving a 70% response rate for each stratum. Each contract in each stratum had a known, nonzero probability of selection. Table 2 presents the number of contracts, the estimated number of state employees, retirees, and adult dependents and the number of respondents for each stratum.

The survey respondent was the person in the household most knowledgeable about the health care received by all family members covered by the employer-sponsored health plan. These respondents constituted our sample. The interviewer used the following screening question to identify this respondent: "For this study, we are interested in speaking to the person who knows the most about the health care received by all of the people in your family covered by your health care plan. Would that be you?" If the respondent indicated that he or she was that person, the interview continued. If not, the interviewer asked the respondent to identify the appropriate person. If necessary, the interviewer arranged a call back to contact this person. The interviewer then began the interview with the above screening question.

The survey contact rates (the number of households contacted divided by the total sample) were 64% for 2001 and 70% for 2002. The survey refusal rates (the number of households that refused to participate divided by the total sample) were 14% for 2001 and 12% for 2002. The survey response rate in both years was 64%. We calculated the response rate using the Council of American Survey Research Organizations method employed for the Behavioral Risk Factor Surveillance System (19). According to this method, the numerator is the number of completed interviews, and the denominator includes all valid households in the sampling frame (i.e., living employees, retirees, and adult dependents, working telephone number, valid health plan membership). This method allocates households with unknown validity (e.g., busy phone line, no answer) to valid or invalid status according to the proportion of valid and invalid households among those with known validity. The final sample included 5609 individuals in 2001 and 6518 individuals in 2002 who subscribed to health plans that were required to provide coverage for SCT.

### Assessing smoking status

There were some differences in the smoking-related questions in the 2001 survey compared with the survey questions in 2002 because the 2001 survey was conducted within a few months of the introduction of the new SCT benefit. In 2001, we included a question to assess smoking status just before the introduction of the benefit. Respondents were asked if they had smoked every day, some days, or not at all during the month of December 2000. This question was used only to estimate the number of individuals who might use insurance coverage for SCT in 2001.

In 2001 and 2002, we asked respondents if they had smoked every day, some days, or not at all during the past 12 months. Individuals were considered smokers if they reported smoking every day or some days. Responses to this question were used to estimate the smoking prevalence rate for the state employee population, to compare estimates between the 2 years, and to estimate the population eligible to use the SCT benefit in 2002.

### Measuring awareness of SCT insurance benefits

We also asked respondents if prescription medications for smoking cessation were covered by their health insurance plan. The question included the list of medications that were actually covered by the DETF's health insurance benefits package. We considered respondents to be aware of coverage if they answered yes to this question.

### Defining users of SCT benefit

We asked self-reported smokers if their health plan had paid for prescription medications to help them quit smoking. We posed a separate question for each of the four prescription medications covered. In 2001, we asked respondents to reply to these questions based on their experience since January 1, 2001, the effective date of the SCT insurance coverage. In 2002, we asked respondents to reply based on their experience during the past 12 months.

### Defining users of OTC medications

In 2002, we asked self-reported smokers if they had purchased OTC medication, such as the nicotine patch or nicotine gum, to help them quit smoking. We defined OTC users as those who reported they had made such a purchase. An individual could potentially report the prescription benefit use, OTC use, or both.

### Data analysis

Analyses consisted of descriptive statistics and tests of the relationships between survey year and each outcome. We used Stata version 8.0 (Stata Corp, College Station, Tex) (20) to perform all analyses, which were weighted for representativeness in the population. To test for differences in demographic characteristics between 2001 and 2002, we used a Pearson chi-square test for variables with multiple response options (age and education) and a Student *t* test for sex. For smoking prevalence, benefit awareness, benefit use, and OTC medication use, we estimated population rates adjusted for the sampling weights*.* For the outcomes assessed in both survey years using comparable survey questions — smoking prevalence and benefit awareness — we used a Student *t* test to test for a difference in population means between 2001 and 2002.

## Results

Table 3 presents the demographic characteristics of the unweighted sample data; Table 4 presents the demographic characteristics of the weighted sample data. We discuss below the weighted sample demographics because our aim is to make inferences to the state employee, retiree, and adult dependent population as a whole. More than 80% of the sample had some college education, and more than 55% of the sample had a 4-year college degree or more. Approximately 44% of the respondents in each year were male. Differences in age, education, and sex for 2001 and 2002 were not statistically significant. We assume that the demographic characteristics of the samples reflect the state employee, retiree, and adult dependent population for each year.

The study outcomes assessed in 2001 and 2002 are presented in Table 5. Population smoking prevalence estimated from the sample declined from 15.6% in 2001 to 13.2% in 2002 (*P* = .01). Awareness of the benefit grew from an initial 20.6% of smokers in 2001 to 27.4% in 2002 (*P* = .02). During the first 3 to 6 months of the benefit's availability in 2001, 7.1% of smokers reported benefit use. In 2002, 13.6% reported benefit use. Benefit use among smokers aware of the benefit was 23.6% in 2001 and 39.6% in 2002. Benefit use among smokers who were unaware of the benefit was 2.9% in 2001 and 3.5% in 2002. (Unaware smokers who reported using the benefit would have paid a typical copayment to receive SCT medications. We assume that they did not interpret use of a copayment as insurance coverage for SCT medications.) 

In 2002, we also collected information on OTC medication use. These findings are presented in Table 6. We found that 9.3% of smokers reported use of the benefit alone (i.e., no OTC medication use). Use of OTC medications alone was reported by 16.9% of smokers (i.e., no benefit use). Few smokers (4.2%) used both OTC medications and the benefit. Of smokers unaware of the benefit, 21.4% reported using OTC medications only to help them quit smoking; 5.2% of smokers aware of the benefit reported using OTC medications only. Among all smokers in 2002, 30.4% reported use of some medication to help them quit smoking (i.e., OTC, benefit, or both).

## Discussion

In a highly educated, working-age population, use of a new SCT benefit was modest among all smokers during its first 2 years of availability. In its second year of availability, only 13.6% of all smokers used the benefit to help quit smoking. However, among smokers who were aware of the benefit, the benefit use rate was higher at 39.6%. These findings are relevant to employers and purchasers in need of population-based benefit-use estimates to introduce their own SCT benefit. For example, 25 state-government employers do not provide an insurance benefit for SCTs to some or all of their employees (9).

There are several potential explanations for the modest benefit-use rates for all smokers identified in this study. Low benefit awareness is one possibility. As described above, the state's promotion of the new benefit was limited. In our study, we found greater benefit use among smokers who were aware of the benefit and greater use of only OTC medications among smokers who were unaware of the benefit. In 2002, 39.6% of smokers aware of the benefit and 3.5% of smokers unaware of the benefit reported using the benefit. In that same year, 21.4% of smokers unaware of the benefit and 5.2% of smokers aware of the benefit reported using OTC medications only to help them quit smoking. Boyle et al also found that smokers aware of the benefit were more likely to use Zyban, one of the covered medications in that study, to make an attempt to quit than smokers who were unaware of the benefit (15). To our knowledge, only one study has examined the effect of a benefit-promotion intervention on smokers' knowledge and use of a SCT benefit. In a randomized controlled trial, Alesci et al found greater benefit knowledge among smokers who received a promotional postcard than those who received notification of the new SCT benefit within annual health plan enrollment materials (21). There was no difference, however, in benefit-use rates between the two groups. Additional research is needed to identify communication strategies that increase benefit awareness and use to inform employers' implementation and promotion of SCT coverage.

Alternatively, perhaps low smoker interest in quitting explains the relatively low rate of benefit use we observed. This explanation, however, seems unlikely. Nationally, 70% of current smokers report that they want to quit smoking (22), and approximately 41% of daily smokers reported making an attempt to quit at least 1 day during the past 12 months (23). In our study, the overall annual SCT use rate (benefit or OTC use) was roughly 30%. This rate implies greater quitting activity than suggested by our annual benefit-use rate of roughly 14%. Future research that examines benefit features such as the products and services covered, their limitations, and how a patient gains access to a benefit may further explain the factors that influence benefit-use rates. Additionally, research that explores smokers' reasons for not using an available SCT benefit would be useful in understanding factors that may increase demand and inform benefit design.

As noted above, annual medication use for SCT in the Wisconsin state employee population, obtained through the insurance benefit or OTC, was roughly 30% of all smokers in 2002. This rate of treatment use is high compared with population-based rates found elsewhere. For example, in 1996, just 20% of smokers who attempted to quit smoking used some form of assistance, self-help, counseling, nicotine replacement therapy, or a combination of these (24). Since that time, the variety and availability of FDA-approved medications for smoking cessation has increased. Results from a 2000 survey found that approximately 25% of privately insured smokers who attempted to quit smoking used a cessation aid (25). The FDA-approved medications assessed in our study can double or triple the likelihood of achieving abstinence from smoking compared with quitting without a cessation aid (3). Our population's rate of SCT use may suggest that smokers are increasingly using these efficacious cessation aides.

Based on our rate of OTC medication use, some may conclude that an SCT benefit is unnecessary or redundant. However, if the objective is to maximize smokers' opportunities to quit, the presence of an SCT benefit may help do just that. It may increase the number of smokers who make an attempt to quit. From our data, we could not determine whether the use of this new benefit may have supplanted the use of OTC medications. Future research should examine the extent to which an SCT benefit increases the number of smokers who make an attempt to quit beyond those who use OTC medications, the number of attempts to quit made beyond those made with the assistance of OTC medications, and the outcomes of those attempts to quit.

Ultimately, the likely value of insurance coverage for SCTs to insurers, employers, and purchasers is its effect on smoking prevalence. Studies have demonstrated a promising link between SCT coverage and improved quit rates (5) and reduced smoking prevalence (4). We observed a significant decline in smoking prevalence in the study population during the first 2 years of the benefit's availability, from approximately 15% to 13%. Low smoking rates are typical among highly educated populations nationally (23) and in Wisconsin (26); thus, the level that we observed is not in itself surprising. However, the decline in smoking prevalence during this short period suggests the need for further research to identify its causes. Caution should be used in linking this decline in prevalence solely to the new SCT benefit as other factors may apply. Our study design did not allow us to isolate the effect of the new SCT benefit on smoking prevalence in the population.

This study has a number of limitations. The sample design was a random sample of employee and retiree contracts for health insurance stratified by plan. The stratification did not extend to the level of sex, age, or education within each plan. Some of these variables have been associated with smoking status (23) and OTC use (27). To the extent that the sampling design did not capture a representative sample of the population, our population outcome estimates for prevalence of smoking and OTC use may be somewhat biased. We are not aware of any published evidence demonstrating an association between sex, age, and educational level with benefit use and benefit awareness.

We made two changes to the survey between 2001 and 2002. First, we altered the reporting period for benefit use. In 2001, we asked respondents to report on benefit use during the first 3 to 6 months of the benefit's availability, beginning with January 1, 2001. In 2002, we asked them to report on benefit use during the previous 12 months. Thus, the 2001 survey reflected 3 to 6 months of SCT coverage, and the 2002 survey reflected 12 months of SCT coverage. Because the benefit-use rates reflect different time periods, they should not be directly compared. Second, we added a question to assess OTC use in 2002.

The survey respondent was the person in the household most knowledgeable about the health care received by all family members covered by the plan. To the extent that such a knowledgeable person is more likely to be aware of and use the SCT benefit, our estimated benefit awareness and use rates may overstate what employers will observe in their insured populations. Future research using claims data to assess benefit use would help to address this potential limitation.

Our study results can help employers overcome a common barrier to the introduction of insurance coverage for SCTs: uncertainty about levels of employee use of a new SCT benefit. We found that approximately 14% of all smokers used the benefit to help quit smoking during its second year of availability. This benefit-use rate provides employers with essential information to estimate the cost of a new SCT benefit. By providing this decision-relevant information to employers, our results may hasten the adoption of national recommendations for insurance coverage for SCTs (1-3). In addition to serving employers' informational needs, these findings may inform public policy more generally as state and federal governments consider implementing the National Action Plan for Tobacco Cessation's recommendation to provide universal coverage for evidence-based SCT medications (28).

## Figures and Tables

**Table 1:** Wisconsin State Employees’ Insurance Benefit for Smoking-Cessation Treatment, Effective January 1, 2001^a^

**Component**	**Cost to Employee**	**Limits**
Coverage includes pharmacological products that by law require a written prescription and are prescribed by a plan provider for the purpose of achieving smoking cessation (i.e., Zyban, nicotine inhaler, spray or patch)	Subject to standard prescription drug co-payment and out-of-pocket maximum	One 3-month course per year
Coverage includes one office visit for counseling and to obtain the prescription	None	One office visit per year

a Source is the Wisconsin Department of Employee Trust Funds (17).

**Table 2:** Sample Strata, Wisconsin Consumer Assessment of Health Plans Survey 2001 and 2002

**Health Plan**	**2001 Sample Strata**			**2002 Sample Strata**		

	**No. Contracts in Sampling Frame**	**Estimated No. State Members**	**No. Respon-** **dents**	**No. Contracts in Sampling Frame**	**Estimated No. State Members**	**No. Respon-** **dents**
1	9,897	21,773	353	9,489	20,876	368
2	8,131	17,888	367	NA^a^	NA	NA
3	16,381	36,038	382	14,948	32,886	377
4	3,876	8,527	339	NA	NA	NA
5	1,138	2,503	287	1,075	2,365	294
6	8,125	17,875	367	7,551	16,612	368
7	1,966	4,325	321	1,869	4,112	325
8	754	1,658	254	721	1,586	274
9	423	930	176	419	922	234
10	1,351	2,972	299	571	1,256	237
11	803	1,766	260	772	1,698	267
12	503	1,106	217	450	990	221
13	408	898	148	379	834	217
14	2,860	6,292	339	2,667	5,867	339
15	3,456	7,603	346	3,057	6,725	345
16	2,547	5,603	334	1,554	3,419	323
17	10,028	22,061	370	8,894	19,567	370
18	7,340	16,148	365	5,943	13,075	365
19	2,997	6,593	341	2,934	6,455	348
20	NA	NA	NA	1,315	2,893	327
21	NA	NA	NA	567	1,247	274
22	NA	NA	NA	631	1,388	290
23	NA	NA	NA	2,935	6,457	367
24	NA	NA	NA	919	2,012	283
Total	82,984	182,559	5,865	69,660	153,242	6,813

a NA indicates that this health plan did not serve the state employee and retiree population in the year listed.

**Table 3:** Unweighted Sample Characteristics, Wisconsin State Employees, Retirees, and Adult Dependents, Wisconsin Consumer Assessment of Health Plans Survey 2001 and 2002

	**Unweighted Sample**	

	**2001** **(N = 5609)** **No. (%)**	**2002** **(N = 6518)** **No. (%)**

**Age, y**		

18-24	144 (2.5)	133 (2.1)
25-44	2217 (40.0)	2407 (37.1)
45-64	2617 (46.7)	3211 (49.5)
≥65	611 (10.8)	733 (11.3)
Total^a^	5589 (100.0)	6484 (100.0)

**Education**		

<High school graduate	95 (1.8)	91 (1.4)
High school graduate or GED	965 (17.2)	1129 (17.4)
Some college	1340 (23.9)	1530 (23.6)
4-year college degree or more	3199 (57.1)	3733 (10.0)
Total^a^	5599 (100.0)	6483 (100.0)

**Sex**		

Male	2589 (46.2)	2986 (45.8)
Female	3020 (53.8)	3532 (54.2)
Total	5609 (100.0)	6518 (100.0)

a Because of missing data, the total number of respondents to this question does not equal the total number of respondents in the analytic sample (i.e., 5609 in 2001 and 6518 in 2002).

**Table 4:** Weighted Sample Characteristics, Wisconsin State Employees, Retirees, and Adult Dependents, Wisconsin Consumer Assessment of Health Plans Survey 2001 and 2002^a^

	**Weighted Sample,** **%**		***P *value^b^**

	**2001**	**2002**	

**Age, y**			

18-24	3.0	2.7	.20
25-44	40.9	38.7	
45-64	46.6	48.6	
≥65	9.5	10.0	

**Education**			

<High school graduate	1.6	1.3	.51
High school graduate or GED	15.9	15.9	
Some college	22.1	23.5	
4-year college degree or more	60.3	59.2	

**Sex**			

Male	44.1	43.9	.86

a The sample data were weighted to be representative of the insured State of Wisconsin employee, retiree, and adult dependent population.

b Pearson’s chi-square test was used to test differences from 2001 to 2002 within the age and education categories; a Student t test was used to test for differences in the sex category.

**Table 5:** Smoking Prevalence, Awareness of Benefit, and Use of Benefit Among Wisconsin State Employees, Retirees, and Adult Dependents, Wisconsin Consumer Assessment of Health Plans Survey 2001 and 2002

	**Sample Population**		***P *value^b^**

	**2001 % (95% CI^a^)**	**2002 % (95% CI)**	
Smoking prevalence	15.6 (14.3-16.8)	13.2 (12.0-14.4)	.01
Awareness of insurance benefit for smoking	20.6 (17.0-24.2)	27.4 (23.1-31.7)	.02

**Use of insurance benefit**			

Among all smokers	7.1 (4.7-9.5)	13.6 (10.2-16.9)	c
Among smokers aware of benefit	23.6 (15.2-32.1)	39.6 (30.3-48.7)	c
Among smokers unaware of benefit	2.9 (0.9-4.9)	3.5 (1.2-5.7)	c

a CI indicates confidence interval.

b The change in mean percentage from 2001 to 2002 was analyzed using a Student t test.

c Benefit use rates are not directly comparable. The 2001 survey was conducted between March and June 2001. 2001 respondents reported on benefit use “since January 1, 2001.” Respondents in 2002 reported on benefit use “during the past 12 months.”

**Table 6:** Over-the-Counter (OTC) Medication and Benefit Use Among Wisconsin State Employees, Retirees, and Adult Dependents Who Smoke, Wisconsin Consumer Assessment of Health Plans Survey 2002

	**Sample Population % (95% CI^a^)**

**Among all smokers**	

Benefit use only	9.3 (6.6-12.1)
OTC use only	16.9 (13.2-20.5)
Benefit use and OTC use	4.2 (2.1-6.4)
Benefit use, OTC use, or both	30.4 (25.9-35.0)

**Among smokers aware of benefit**	

OTC use only	5.2 (2.4-8.0)

**Among smokers unaware of benefit**	

OTC use only	21.4 (16.7-26.2)

a CI indicates confidence interval.
